# The effect of *Triticum sativum* (wheat) germ on postpartum pain: A double-blind clinical trial

**DOI:** 10.22038/AJP.2021.18179

**Published:** 2021

**Authors:** Samira Mehravar, Sedigheh Amir Ali Akbari, Malihe Nasiri, Faraz Mojab, Hajar Abbasi

**Affiliations:** 1 *Department of Midwifery & Reproductive Health, School of Nursing and Midwifery, Shahid Beheshti University of Medical Sciences, Tehran, Iran*; 2 *Midwifery and Reproductive Health Research Center, Department of Midwifery and Reproductive Health, School of Nursing and Midwifery, Shahid Beheshti University of Medical Sciences, Tehran, Iran*; 3 *Department of Basic sciences, School of Nursing and Midwifery, Shahid Beheshti University of Medical Sciences, Tehran, Iran*; 4 *Department of Pharmacognosy, School of Pharmacy, Shahid Beheshti University of Medical Sciences, Tehran, Iran*; 5 *Preventative Gynecology Research Center, Shahid Beheshti University of Medical Sciences, Tehran, Iran*

**Keywords:** Postpartum pain, Triticum sativum (wheat) germ Complementary medicine Medicinal plants

## Abstract

**Objective:**

Postpartum pain (PP pain) is a common problem after vaginal delivery. Some herbs are used to reduce PP pain. Due to the anti-inflammatory properties of *Triticum sativum* (wheat) germ, this study was conducted to investigate the effect of wheat germ on PP pain.

**Materials and Methods:**

This is a randomized, double-blind, placebo-controlled clinical trial performed on 90 women who had a vaginal delivery and complained of moderate to severe PP pain. The participants were randomly divided into two groups. In the intervention group, a capsule containing 500 mg of wheat germ was taken every 6 hr for 2 days and in the control group, a placebo capsule was taken in the same order. The severity of PP pain was measured before and one hour after receiving the capsule by using the Visual Analogue Scale.

**Results:**

The two groups were not different in terms of pain severity before the intervention. The PP pain in women with moderate pain was significantly reduced in both groups, the reduction was greater in the wheat germ group (GEE=0.04) but this reduction was not significant. The PP pain in women with severe pain was significantly reduced in both groups, however, the reduction was significantly greater in the wheat germ group (GEE=0.63, p=0.007). Moreover, the results showed that the use of mefenamic acid in the wheat germ group was significantly lower than the control group (p=0.04). Moreover, no side effect was reported after consuming the wheat germ.

**Conclusion:**

It seems that wheat germ reduces severe PP pain. Further research on this plant is recommended.

## Introduction

The anatomical, physiological, and hormonal adaptations of the female body to pregnancy are extensive, and most of these adaptations return to their pre-pregnancy state after delivery. Postpartum pain (PP pain) is one of the most important and common problems caused by this return process during postpartum period (Dashe et al., 2018 ▶; Murphy et al., 2018 ▶) and affects millions of young women (Pourmaleky et al., 2013 ▶; Gosiewski et al., 2019 ▶). When placenta and membranes are removed in the third stage of labour, the uterus contracts to keep the large uterine arteries contracted and prevent postpartum hemorrhage (Dashe et al., 2018 ▶). As a result of these contractions, chemical mediators such as bradykinin, leukotrienes, prostaglandins, serotonin, and lactic acid are released and they cause pain (Pourmaleky et al., 2013 ▶; Fahey, 2017 ▶). It seems that the release of prostaglandins which causes uterine contractions, is the main reason of PP pain (Kheiriyat et al., 2016 ▶; Holdcroft et al., 2003 ▶). PP pain is felt in lower abdomen and lower back similar to labour pain. In primiparous women, the uterus usually remains contracted after delivery whereas in multiparous women, the uterus contracts sharply at different intervals (Dashe et al., 2018 ▶). The severity of PP pain is described from similar to menstrual cramps with severe discomfort to worse than labour pain (Kheiriyat et al., 2016 ▶; Holdcroft et al., 2003 ▶). This pain usually lasts 3 to 4 days and sometimes up to a week after delivery and increases with the number of deliveries (Dashe et al., 2018 ▶; Fahey, 2017 ▶).

During breastfeeding, when the baby sucks the mother's breast, due to the release of oxytocin from the posterior pituitary gland, the areola smooth muscles contract, leading to milk secretion. Oxytocin also causes the smooth muscles of the uterus to contract and consequently, the mother would suffer from more severe pain (Dashe et al., 2018 ▶). Pain and stress can reduce oxytocin secretion from the posterior pituitary gland by increasing adrenaline. Therefore, in addition to the mother's discomfort, PP pain can impede the early onset of breastfeeding by disrupting the oxytocin reflex and flow of breast milk. This can lead to the mother’s inability to breastfeed and reduce her attention to the baby, thus disrupting their communication (Pourmaleky et al., 2013 ▶; Fahey, 2017 ▶).

According to previous studies, 77% of women experience PP pain (Pourmaleky et al., 2013 ▶). Recent findings suggest that there are various methods for relieving PP pain including massage therapy, reflexology, heat therapy, relaxation, skin stimulation, herbal medicine, and chemical drugs (Fahey, 2017 ▶; Kheiriyat et al., 2016 ▶; Nia et al., 2019 ▶; Afravi et al., 2019 ▶). The most common method for relieving PP pain is the use of oral analgesics such as mefenamic acid, ibuprofen, and acetaminophen (Pourmaleky et al., 2013 ▶; Afravi et al., 2019 ▶). Despite the great effect of these analgesics in terms of pain reduction, their side effects include nausea, vomiting, diarrhea, abdominal pain, gastrointestinal bleeding, dizziness, vertigo, and drowsiness (Fahey, 2017 ▶).

Nonsteroidal anti-inflammatory drugs (NSAIDs) such as mefenamic acid by restraining the cyclooxygenase, inhibit the conversion of arachidonic acid to prostaglandin which is the main reason for PP pain (Kheiriyat et al., 2016 ▶). Due to the side effects of chemical drugs, currently, medicinal plants have become more important and people are attracted to them as one of the most common treatments for diseases. In addition, Medicinal plants are more cost-effective compared to chemical drugs (Niazi et al., 2019 ▶).

Among medicinal plants, wheat germ (*Triticum sativum*) contains nutritious and valuable components. It has anti-inflammatory, antioxidant, sedative, anti-depressant, and nerve-relaxing properties. It is effective in improving various diseases and health problems such as cancer, obesity, diabetes, asthma, anemia, eczema, hair loss, hypertension, colic, ulcers, gastritis, schizophrenia, migraine, ataxia, nervous system diseases, and acute mental illnesses (Singh et al., 2012 ▶; Jeong et al., 2017 ▶; Karami et al., 2019 ▶; Kumar et al., 2016 ▶).

Wheat germ has many types of minerals, vitamins, and proteins. It also contains magnesium, zinc, calcium, selenium, sodium, potassium, phosphorus, chromium, vitamins D, A, E, B1, B2, B3, B6, and B12, folic acid, iron, essential fatty acids, enzymes, and antioxidants (Kumar et al., 2016 ▶; Atallahi et al., 2014 ▶; Ataollahi et al., 2015 ▶; Moradi et al., 2017 ▶; Morovvati et al., 2018 ▶; Hadijafari and Morovvati,2019 ▶).

Wheat germ activates neuropeptides, cytokines, and macrophages. It can reduce inflammation by decreasing the level of prostaglandins. The most important compounds in wheat germ that can have a great effect on relieving uterine smooth muscle contraction include vitamins B, E, and D, magnesium, and calcium (Atallahi et al., 2014 ▶; Zakaria et al., 2017 ▶).

Various studies have reported the positive effects of wheat germ components (vitamins B6, E, and D, calcium, magnesium, and essential fatty acids) on reducing prostaglandins levels and the severity of menstrual cramps. The study of Randabunga et al. (2018) ▶ showed the positive effect of vitamin B6 on restlessness. In addition, Charandabi et al. (2017) ▶ confirmed the positive impact of calcium supplements on decreasing the intensity of menstrual pain. Ozel et al. (2019) ▶ reported the positive effect of vitamin D on reducing prostaglandin production and dysmenorrhea.

In the study of Kashanian et al. (2013) ▶, the positive impact of vitamin E on increasing internal opioids and reducing pain intensity was reported. Atallahi et al. (2014) ▶ also confirmed the effectiveness of wheat germ extract on decreasing the severity of menstrual pain due to the presence of components such as vitamins E, B6, and B1, zinc, and calcium. The study of Hosseinlou et al. (2014) ▶ revealed the effectiveness of vitamin B1 tablets on the severity of menstrual pain by affecting the neuromuscular system.

Considering the positive effect of wheat germ and its compounds on reducing prostaglandins production, increasing internal opioids and thus reducing menstrual pain, and the similar mechanism of uterine spasm during menstruation and postpartum, we investigated the effect of wheat germ on PP pain in the present study.

## Materials and Methods

The present study was a double-blind clinical trial conducted after the approval of the Ethics Committee of Shahid Beheshti University of Medical Science with code (IR.SBMU.PHARMACY.REC.1399.128) and registration in the Clinical Trial Centre with code (IRCT20200211046452N1).

**Preparation of drug and placebo**

The wheat germ capsules contained 500 mg of wheat germ powder and they were obtained from Golha Company (Iran) which was approved by Iran Ministry of Health and Medical Education. This dose was determined based on study of Atallahi et al. 2014 ▶, which showed the effect of wheat extract on dysmenorrhea. The placebo capsules contained starch powder. Both capsules were made in the pharmacognosy laboratory at the school of pharmacy at Shahid Beheshti University of Medical Sciences. They had a completely similar appearance and they were placed in similar packages. Packages were coded with A or B by the pharmacist. Mefenamic acid 250 mg capsules were made by Razak Pharmaceutical Company.

**Sample size**

The minimum number of sample size was calculated based on a similar study (Chananeh et al., 2018 ▶) through the following formula:



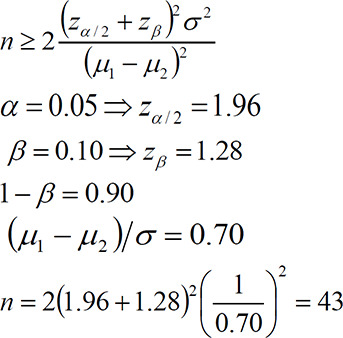



Based on this formula, at least 43 subjects were estimated for each group. Considering 5% probability of sample reduction, 45 subjects were estimated for each group. 

**Study participants**

The participants have been selected for 6 months based on the following inclusion criteria:

 originally Iranian, literate, live with spouse, age of 18-35 years, vaginal delivery with cephalic presentation without using tools (vacuum/forceps), epidural or spinal anaesthesia, first or second delivery, singleton and healthy infant with 37-42 weeks of age, infant’s weight of 2.5-4 kg, exclusive breastfeeding, spontaneous delivery of placenta and membranes, complain of moderate to severe after-pain two hours after delivery, absence of third- or fourth-degree perineal lacerations, not using any drugs to relieve pain within 24 hr prior to delivery, not being narcotic addict, no underlying diseases (such as diabetes, celiac, hypertension, heart disease, infectious diseases, etc.), no mental diseases, no sensitivity to wheat germ or other herbal medicines, no sensitivity to NSAIDs (mefenamic acid, indomethacin or ibuprofen) and no history of C-section or abdomino-pelvic surgery. 

The exclusion criteria in this study was as follows: serious maternal complications after delivery including (postpartum hemorrhage, fever above 39°C and blood pressure above 140/90 mmHg), consumption of any herbal medicines or chemical drugs other than mefenamic acid to relieve pain, lack of breastfeeding, unwillingness to continue participating in the study and sensitivity to wheat germ.

A member of the research team who was responsible for collecting information continuously referred to the obstetrics ward of Mahdieh hospital in Tehran, Iran to choose the qualified participants. Then, she explained and clarified the objective, importance, and procedure of the study to those who were eligible to fill the questionnaires. In the first step, the participants signed the written consent and they were assured that their information is confidential. It was also clarified that they could leave the study at any time. The data were collected through demographics and obstetrics questionnaires, the Visual Analogue Scale (VAS), and the inventory of wheat germ capsule side effects. They were completed through observation and interview. 

Content validity was used to determine the validity of demographics and obstetrics questionnaires and the inventory of wheat germ capsule side effects. The initial forms were prepared according to the research objectives after reviewing related articles and reference books. These forms were approved by 10 members of the midwifery faculty of Shahid Beheshti University of Medical Sciences. The Visual Analogue Scale (VAS) was used to measure pain intensity. It is a standard scale worldwide and its validity and reliability have been confirmed in previous studies (Cheatham et al., 2018 ▶; Pathak et al., 2018 ▶; Thong et al., 2018 ▶).

Out of 152 women who were assessed at the beginning, 104 were eligible to enter the study according to the inclusion criteria. They were assigned to group A or group B using the random number function in Excel. According to the random code of the subject, the capsule was given to the mother from a package with the same code. The research team and participants were unaware of whether the capsules contain wheat germ or starch powder.

Two hours after delivery, the severity of PP pain was measured and recorded using VAS which is a 10 cm horizontal ruler numbered from 0-10. On this scale, zero means ‘no pain’, 1-3 ‘mild pain’, 4-6 ‘moderate pain’, and 7-10 ‘severe pain’. 

Participants who complained of moderate to severe PP pain with a score of 4 or above, were selected. They were then given a wheat germ capsule or placebo every 6 hr for 48 hr. The severity of after-pain was measured and recorded in both groups before and one hour after taking the capsules. If the mothers’ PP pain was not relieved one hour after taking capsules, she would have taken a capsule of mefenamic acid 250 mg as the common treatment of PP pain. The number of mefenamic acid capsules was also recorded and mothers were informed that the consumption of any analgesics except mefenamic acid was banned. Participants were taught how to use the capsules and how to measure pain severity. In order to ensure the correct recording of pain measurements by the participants, all cases were recorded 4 times for PP pain severity in the presence of a member of the research team. After that, the participants were asked to record their pain until the end of the study. The condition of all participants was monitored by telephone for 2 days and they were reminded to use the capsule and record all information related to any side effects or problems.

**Statistical analysis**

Data were analyzed using SPSS (version 20) software. Chi-square and Mann-Whitney tests were used to compare the qualitative variables between the two groups. Quantitative variables were evaluated by Two Independent Sample T-test and GEE (Generalized Estimating Equation) test. In all tests, a p<0.05 was considered statistically significant. 

## Results

**Figure 1:**
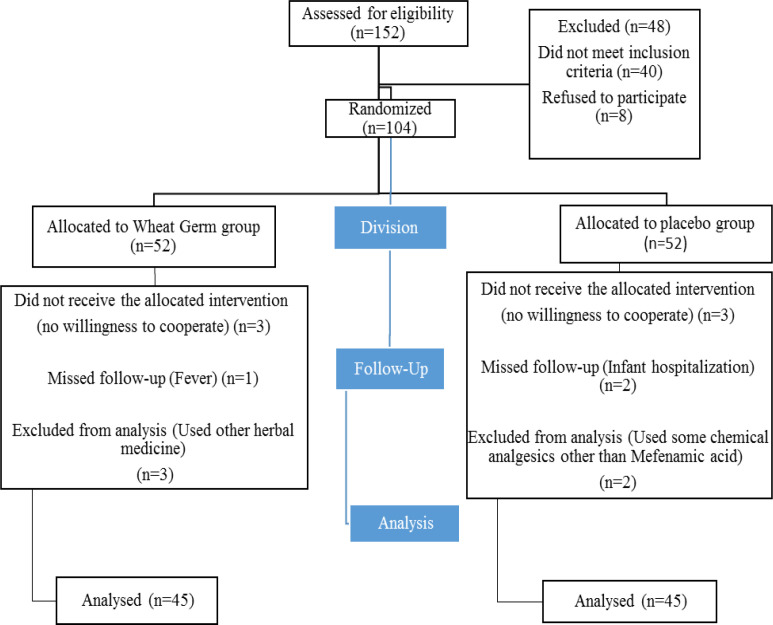
Flow of participants through the research

In this trial, 52 participants were included in each group. In the group taking wheat germ capsules, 3 participants because of using other herbal medicines, 1 participant because of having fever above 39°C and 3 participants because of unwillingness, were excluded from the study. Finally, 45 participants were analysed in this group. In the group taking placebo, 3 participants due to unwillingness, 2 participants due to infant hospitalization and lack of breastfeeding and 2 participants due to the consumption of pain killers other than mefenamic acid, were excluded, eventually 45 participants were analyzed in this group (Figure 1).

Based on the collected information, the two groups did not significantly differ in terms of individual variables including age, education, occupation of the subject and spouse, and the economic status of the family. Also, the two groups were homogeneous in terms of obstetrics variables including the number of pregnancies, the number of deliveries, gestational age, duration of the active phase of the first stage of labor, duration of the second stage of labor, birth weight, head circumference at birth, having an episiotomy, receiving oxytocin during labor and postpartum, infant’s sex, pregnancy desirability, and infant’s sex desirability and there was no statistically significant difference between the two groups (Table 1).

**Table 1:** Comparison of demographic and obstetrics characteristics in the two groups

p value	Placebo N=45	Wheat germ N=45	Groups	
			Variables	
0.93**	26.51±4.19*	26.57±4.03*	Age (year)	
0.34***	20 (44.4%)	25 (55.6 %)	High school and lower	Education
	18 (40 %)	14 (31.1%)	Diploma	
	7 (15.6 %)	6 (13.3%)	University	
0.309***	42 (93.3%)	44 (97.8%)	Housewife	Occupation
	3 (6.7%)	1 (2.2%)	Employee	
0.669***	17 (37.8%)	16 (35.6 %)	High school and lower	Husband’s Education
	23 (51.1%)	22 (48.9%)	Diploma	
	5 (11.1%)	7 (15.6%)	University	
0.405***	14 (31.1%)	18 (40%)	Worker	Husband’s Occupation
	11 (24.4%)	10 (22.2 %)	Employee	
	20 (44.4%)	17 (37.8 %)	Self – employment	
0.81****	34 (75.6%)	33 (73.3%)	Yes	Wanted pregnancy
	11 (24.4%)	12 (26.7%)	No	
0.64****	30 (66.7%)	32 (71.1%)	Yes	Episiotomy
	15 (33.3%)	13 (28.9%)	No	
0.09***	15 (33.3%)	23 (51.1%)	One	Number of deliveries
	30 (66.7%)	22 (48.9%)	Two	
0.405**	38.48±1.03	38.51±1.12	Duration of pregnancy(week)	
0.661**	3.22±3.71	3. 55±3.47	Oxytocin injected during labor(unit)	
0.633**	34.88±6.26	35.55±6.92	Oxytocin injected after delivery(unit)	
0.613**	169.66±31.55	173.55±40.58	Duration of the First stage of labor(minute)	
0.06**	54.11±13.02	48.66±14.03	Duration of the second stage of labor(minute)	

*Mean±Standard Deviation, **Two Independent Sample T-Test, *** Mann-Whitney Test, ****Chi-Square.

**Figure 2:**
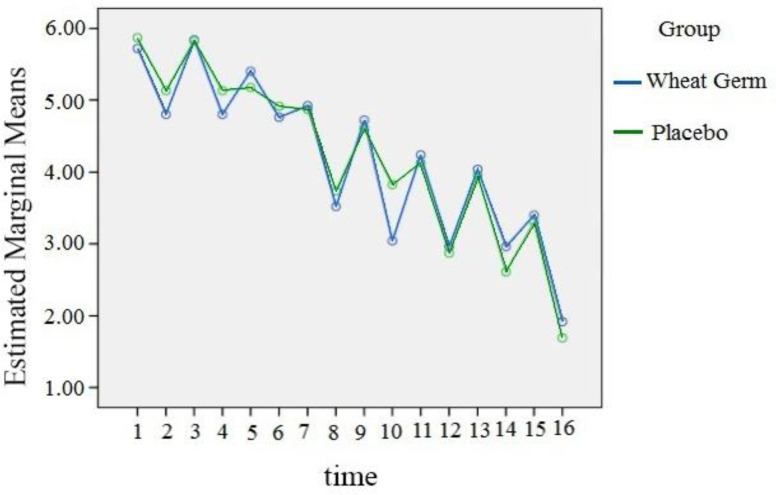
Estimated marginal mean graph of pain severity in moderate pain class

**Figure 3:**
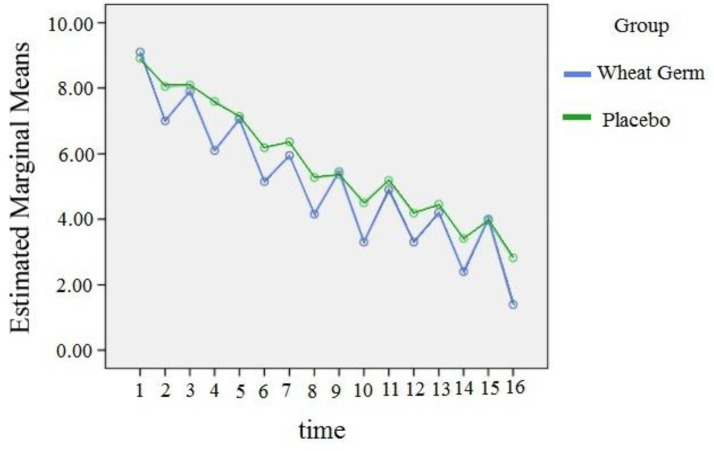
Estimated marginal mean graph of pain severity in severe pain class

**Figure 4:**
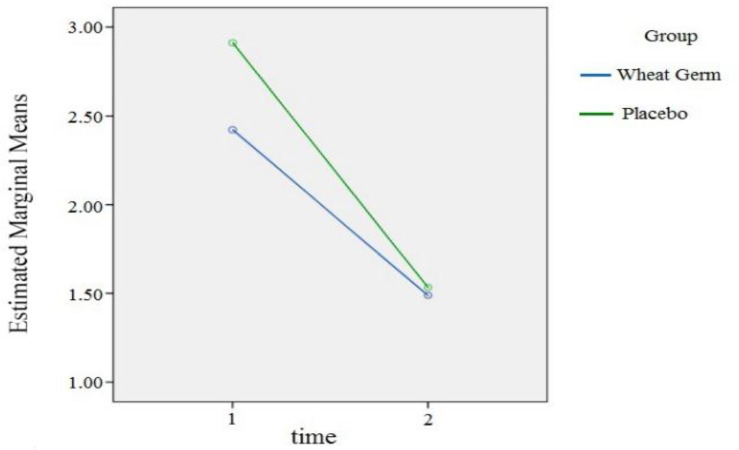
Estimated marginal mean graph of Mefenamic acid taken in the two groups

The two groups were not different in terms of pain severity before the intervention. In the next step, the pain intensity in the two groups was separately compared between the moderate and severe classes. Due to the fact that the data did not have a normal distribution, the GEE model was used to analyze them. In moderate class, the pain intensity in wheat germ group was 0.038 less than control group, but this difference was not significant. [in wheat germ group n=25(55.6%) and in placebo group n=22(55.1%)]. However, in case of severe class, the pain intensity in wheat germ group was 0.632 less than the control group and this difference was statistically significant (p=0.007) (Table 2). Figures 2 and 3 show the estimated marginal mean of pain severity for the moderate and severe classes, respectively. It is worth pointing out that the pain severity was measured 16 times in this study. The mean number of mefenamic acid capsules taken during the two days was lower in the wheat germ group (mean: 3.9) than the placebo (mean: 4.4). The difference in the number of mefnamic acid taken between the two groups was statistically significant (p=0.04) (Table 3). The marginal mean of mefenamic acid intake for both groups during the two days is presented in Figure 4. Furthermore, no side effects such as abnormal bleeding, nausea, vomiting, headache, diarrhea, fever, urticaria, pruritus, respiratory problems or dizziness were observed in either group.

**Table 2:** GEE (Generalized Estimating Equation) model results for comparison of the two groups in the moderate and severe pain

Intensity of Pain	Parameter	B	Std. Error	95% Wald Confidence Interval		Hypothesis Test	
				Lower	Upper	Wald Chi-Square	p-value
moderate	(Intercept)	6.052	.1779	5.703	6.400	1157.365	.000
	[Group=Wheat Germ]	-.038	.2228	-.475	.398	.029	.864
	[Group=Control]	0a	.	.	.	.	.
	Time	-.215	.0115	-.237	-.192	347.827	.000
severe	(Intercept)	8.973	.1825	8.616	9.331	2417.929	.000
	[Group= Wheat Germ]	-.632	.2349	-1.092	-.171	7.226	.007
	[Group=Control]	0a	.	.	.	.	.
	Time	-.383	.0204	-.423	-.343	353.155	.000

**Table 3:** Comparison of mefenamic acid capsule taken during 48 hours after delivery in the two groups

Groups	Wheat Germ N=45	Placebo N=45	p value
Number of Mefenamic acid capsules consumed			
2	6 (13.3%)	3 (6.7%)	0.04*
3	9 (20%)	6 (13.3%)	
4	15 (33.3%)	11 (24.4%)	
5	11 (24.4%)	18(40%)	
6	4 (8.9%)	7 (15.6%)	

*Mann-Whitney Test

## Discussion

The results of the present study showed that wheat germ significantly reduces the severity of severe PP pain. There are no studies that directly examine the effect of wheat germ on PP pain. Some studies demonstrated the effect of wheat germ and some of its important compounds (including vitamin D, B, and E, calcium, magnesium and zinc) on the reduction of prostaglandins production and thus reduction of the uterine smooth muscle contractions and uterine cramping pains (Vilvapriya and Vinodhini, 2018 ▶; Charandabi et al., 2017 ▶; Moini et al., 2016 ▶; Atallahi et al., 2014 ▶). Since the mechanism of uterine contractions in PP pain is similar to primary dysmenorrhea and its main stimulus seems to be the production and release of prostaglandins, the results of these studies can be a good justification for our findings.

In a clinical trial conducted by Atallahi et al. (2014) ▶, daily consumption of wheat germ extract from day 5 to 16 of the menstrual cycle for two months, resulted in reduction of uterine smooth muscle contractions and thus significantly reductions in the severity of primary dysmenorrhea and associated systemic symptoms including headache. Also, in another study by Ataollahi et al. (2015) ▶ on the effect of wheat germ extract on the premenstrual syndrome, daily consumption of wheat germ extract for two months significantly reduced sensitivity, tension, pain, tenderness of the breasts and headache. Wheat germ has anti-inflammatory properties and activates macrophages, neuropeptides, cytokines and thus reduces inflammation and pain (Jeong et al., 2017 ▶). These results confirm the findings of our study.

Vitamin E is an antioxidant and prevents the production of arachidonic acid and prostaglandins, thus reducing uterine smooth muscle contractions and pain (Vilvapriya and Vinodhini, 2018 ▶). Numerous studies have shown the positive effect of vitamin E on primary dysmenorrhea (Kashanian et al., 2013 ▶; Pakniat et al., 2019 ▶). In the study of Vilvapriya and Vinodhini (2018) ▶, consumption of vitamin E significantly reduced the duration and severity of pain in patients with primary dysmenorrhea. The positive effect of wheat germ on PP pain may be due to its vitamin E content which is in line with the present study. 

In the study of Randabunga et al. (2018) ▶, the positive effect of vitamin B6 on reducing prostaglandins level and pain intensity of primary dysmenorrhea was observed which is in line with the present study.

Several studies indicated that vitamin D consumption reduces muscle pain and improved life quality (Gendelman et al., 2015 ▶; Ghai et al., 2017 ▶). In the study of Abbasi et al. (2012) ▶, it was found that treatment with vitamin D was effective in relieving musculoskeletal pain in people with vitamin D deficiency. In another study, the positive effect of vitamin D on reducing the pain of breast cancer was observed (Khan et al., 2010 ▶). Also, in various clinical trials, the effect of vitamin D on reducing pain in uterine smooth muscles and dysmenorrhea was mentioned (Cheatham et al., 2018 ▶; Moini et al., 2016 ▶; Haghighian, 2019 ▶). Wheat germ contains vitamin D. Hence, the effect of wheat germ on PP pain may be due to the effect of vitamin D on the uterine smooth muscles and reduction of contractions which confirms our findings.

Wheat germ is rich in calcium. Calcium reduces pain (Hosseinlou et al., 2014 ▶; Zarei et al., 2017 ▶). Also, in a clinical trial conducted by Mehrpooya et al. (2017), consumption of calcium and fish oil reduced the severity of primary dysmenorrhea. Since wheat germ contains calcium, the positive effect of wheat germ on PP pain may be due to its calcium content which is in line with the present study.

The severity of PP pain also decreased in the placebo group which is probably due to the psychological effects of the drug. The results of Atallahi et al. (2014) ▶ showed that the placebo had a positive effect on reducing pain.

In the present study, use of mefenamic acid in the intervention group was significantly less than the placebo group. The results of Atallahi et al. (2014) ▶ also showed that wheat germ consumption significantly reduced the number of analgesics taken by the intervention group.

Based on the results, no abnormal bleeding or side effects such as nausea, vomiting, headache, diarrhea, fever, urticaria, pruritus, respiratory problems or dizziness were observed in either group. 

The wheat germ reduced the intensity of after-pain especially in case of severe pain. It was shown that the group taking wheat germ capsules consumed less mefenamic acid compared to the placebo group. Moreover, no side effect was reported after consuming the wheat germ. Further research on this plant is recommended. 
